# The 2D:4D Marker and Different Forms of Internet Use Disorder

**DOI:** 10.3389/fpsyt.2017.00213

**Published:** 2017-11-20

**Authors:** Marko Müller, Matthias Brand, Julia Mies, Bernd Lachmann, Rayna Yordanova Sariyska, Christian Montag

**Affiliations:** ^1^Institute of Psychology and Education, Ulm University, Ulm, Germany; ^2^Department of General Psychology: Cognition, University of Duisburg-Essen, Duisburg, Germany; ^3^Key Laboratory for Neuroinformation, Center for Information in Medicine, School of Life Science and Technology, University of Electronic Science and Technology of China, Chengdu, China

**Keywords:** 2D:4D marker, digit ratio, androgen, prenatal testosterone, Internet addiction, problematic Internet use, Internet gaming disorder, Internet use disorder

## Abstract

Internet use disorder (IUD) presents a growing problem worldwide. Among others, it manifests in loss of control over Internet usage and social problems due to problematic Internet use. Although IUD currently is not an official diagnosis in DSM-5 or ICD-10, mounting evidence suggests that IUD indeed could be categorized as a behavioral addiction. On a systemic neuroscientific level, IUD is well characterized and dysfunctions in the fronto-striatal-limbic loop have been observed in persons being afflicted with IUD. On a molecular level underlying these neural dysfunctions less is known. Therefore, the present research investigates the influence of prenatal testosterone as measured *via* the 2D:4D marker of the hand on IUD. Testosterone represents an interesting hormonal marker, because sex differences in IUD have been observed, e.g., males show higher tendencies toward Internet gaming disorder (IGD) or females toward overusage of online social networks (both compared to the contrary sex). In *N* = 217 participants associations between the 2D:4D marker of the hand and both unspecified IUD and specific forms of IUD were investigated. It appeared that more female hands (right side; characterized by higher digit ratio of the index to the ring finger, i.e., >1, meaning lower prenatal testosterone) were associated with lower IGD (*rho* = −0.17, *p* = 0.01, *N* = 211). This effect was driven by the facet of loss of control of Internet Gaming in the whole sample (*rho* = −0.20, *p* < 0.01, *N* = 211) and the female subsample (*rho* = −0.20, *p* = 0.02, *N(f)* = 137). Aside from this, a negative association appeared between the facet of loss of control of generalized IUD and the right digit ratio in males underlining earlier work. In sum, the present work demonstrates that the 2D:4D marker is an interesting marker for Internet addiction and can be easily included as a biomarker to understand the biological underpinnings of Internet (over-)usage.

## Introduction

Currently, about 3.75 billion users of the world population are online.^1^ Being online provides users with manifold opportunities to stay in touch with people over long-distance, communicate easily and find information quickly as long as a smartphone/Internet signal is available. Despite these positive effects of growing digital worlds, more and more researcher worldwide discuss if excessive use of digital channels might reflect addictive behavior [see review by Ko et al. (1); see compendium by Montag and Reuter (2)].

Different terms have been suggested to describe excessive online usage including *compulsive Internet use, problematic Internet use, Internet addiction*, and due to recent advances in DSM-5 also *Internet use disorder* (IUD). IUD was coined in line with the inclusion of the term Internet gaming disorder (IGD) in DSM-5 in its appendix (3, 4). IUD can be described by symptoms such as loss of control over one’s own Internet usage, problems in private and business life due to excessive use, withdrawal symptoms when not being online and development of tolerance, to name a few. Although researchers need to be careful not to overpathologize everyday life (5), mounting evidence suggests that digital overusage in its extreme forms indeed could pose dramatic problems as also underlined by some drastic news reports from Asia [including death cases; e.g., see Ref. (6)]. Prevalence rates of IUD vary across the world with Asian countries (7) being more afflicted than Western countries. In Germany, about 1% of the population is addicted to the Internet according to representative numbers from the PINTA study (8).

The last years have seen a vivid scientific discussion on the nature of IUD (2), in particular also if there exists a generalized IUD, which needs to be contrasted with specific forms of IUD (9). Generalized IUD^2^ refers to general overusage of many online channels and spending too much time in digital worlds, whereas specific forms of IUD rather exclusively describe persons who overuse one online-channel such as shopping, gaming, pornography, gambling, and social networks (hence online communication). With the diversity of available online channels also the prevalence of IUD in males and females changed. Whereas in the early years of the Internet, Internet usage, and related addictive behavior was more associated with being male, things have dramatically changed in the last years. Whereas online shopping (10) and online social networking channels (11) are more associated with being female, overusage of platforms being related to pornography (12), Internet gaming (13), or online gambling (14) are more a domain of males.

Internet use disorder has been characterized well on a systemic neuroscientific level with dysfunctions of the fronto-striatal-limbic loop as also seen in other forms of substance-dependent addictive-behavior (15, 16). On a molecular level, much less is known. Clearly, the molecule dopamine represents an important transmitter system in the brain to explain craving for online content, when a person afflicted is confronted with a relevant online cue. This mirrors in striatal (over-)activity in fMRI setup, e.g., when online gamers are confronted with stills from the favorite game in a brain scanner (17). It is well known that the striatal region occupies a high number of D_2_ receptors [e.g., Ref. (18)]. Persons suffering from IUD have been associated with lower D_2_ receptors (19, 20), something also observed in alcoholics (21, 22). Aside from dopamine, also the transmitter systems of serotonin (23) and acetylcholine (24) have been implicated in playing a role for IUD.

One of the most recent models to understand specific forms of IUD represents the I-PACE model of Brand et al. (3) targeting the interaction of person, affect, cognition, and execution variables to understand IUD. This model explicitly states among the person variables *biology* to play an important role to understand the genesis and maintenance of specific types of IUD. This has been also underlined by heritability estimates derived from twin studies in the last years [e.g., Ref. (25, 26)]. A roadmap to study the molecular basis of IUD was missing until recently. To close this gap, Montag et al. (27) published an affective neuroscience framework. In this context, an easy and rather obvious candidate to understand IUD might be the steroid hormone testosterone (before testing the many candidates as proposed in Montag’s model). Given the often-observed sex-dimorphism in the different forms of IUD as presented above, sex steroids might be crucial to understand individual differences in IUD from the perspective of a molecular psychologist.

An easy way to assess biological marker giving insights into prenatal testosterone represents the 2D:4D marker of the hand [see overview in Ref. (28)]. Prenatal sex steroids, such as its well-known representative testosterone, regulate brain structure and function (29) as well as finger growth during embryogenesis [again see, Ref. (28)]. The 2D:4D marker is assessed via measuring the length of the index (second digit—2D) to the length of the ring finger (fourth digit—4D; see also Section “Materials and Methods”). It has been demonstrated that female hands are usually characterized by higher digit ratios (hence longer index to ring finger) compared to male hands (characterized by lower digit ratios—hence longer ring compared to index finger). This effect is in particular pronounced for the right hand (30), although it is still not clear why this is the case. Moreover, and of importance to note, also males with more female hands, and females with more male hands can be observed in the population. The 2nd to 4th finger ratio is stable over life [Manning et al. (31); see also evidence from fetal development by Malas et al. (32)]. Different lines of arguments have been put forward to understand why the digit ratio of the hand indeed could represent an indirect marker for the prenatal (but not actual) testosterone level (33). Among these are direct links between the 2D:4D ratio to prenatal testosterone levels, but most pronounced in its link to prenatal testosterone to estradiol ratios (34). In addition, evidence comes from molecular genetic association studies linking a polymorphism of the androgen receptor gene to individual differences in the 2D:4D ratio (35). For more details, see Manning (28).

The 2D:4D marker has been investigated in the realm of many different research areas, in particular those where a sex-dimorphism can be observed (such as that more males than females are affiliated with a certain condition and vice versa). As examples, lower 2D:4D ratios have been associated with autism (36, 37), and higher 2D:4D ratios associate with schizophrenia (38); see also links with schizotypal personality traits as presented by Zhu et al. (39). The 2D:4D marker has been investigated in the context of stuttering (40) and further relationships between the 2D:4D marker and psychological/behavioral phenotypes were identified, such as lower 2D:4D finger ratios account for reproduction success and dominance (41), number of life-time sexual partners (42) and neuroticism (43, 44) as well as cooperativeness whereas aggression appeals lead to more pro-social behavior for high 2D:4D ratios (45, 46). Smaller index-to-ring-finger ratios (hence more male hands) have also been linked to personal qualities and characteristics such as athletic performance (47, 48), spatial abilities (49–52), abstract reasoning (53), and numeric abilities (54–56).

Recently, the 2D:4D ratio of the hand was also investigated in the context of IUD. Lower 2D:4D values of the right hand (which means more typical male hands with longer ring finger compared to index finger) in males were associated with unspecified IUD (57), an effect potentially driven by a specific form of IUD—namely IGD—which was not assessed in this work [only the 20 item Internet Addiction Test (IAT) was administered]. In line with this idea, Kornhuber et al. (58) demonstrated lower 2D:4D ratio values in young males diagnosed with video game addiction, compared to healthy controls. Deriving from these first results, higher prenatal testosterone could represent a vulnerability factor to develop IUD, in particular in males.

As these first works did not address potential associations between 2D:4D and the manifold specific forms of IUD, we aimed with the present research to answer several questions. First of all, we aimed at the replication of the finding that lower digit ratios would predict higher tendencies toward IGD, in particular in males (but perhaps also females). Finally, with this study, it is the first time that data on possible associations between other specific forms of IUD and the digit ratio are presented. For IUD in the realm of online activities being more visible in females (shopping, social networking), we expected more female hands (hence higher digit ratios), for the remaining online channels (pornography, gambling), we expected higher tendencies toward IUD being more associated with more male hands (lower 2D:4D ratio). As Montag et al. (59) demonstrated that Internet communication disorder (ICD) highly overlaps with generalized/unspecified IUD, we aimed with the present sample to revisit the question how different forms of IUD are linked to each other. Given that this earlier work could not address the question if such associations are independent of gender, we present this kind of data in the supplement for further studies.

## Materials and Methods

### Participants and Sociodemographic Characteristics

For this study, we used data collected from November 2016 till May 2017 of *N* = 217 participants from the Ulm Gene Brain Behavior Project (UGBBP). The mean age of our sample was M = 23.41 years (SD = 7.77) and consisted of 77 men and 140 women. Most of them (83.9%) were students (182). A total of 204 participants had German (94.0%) as a mother tongue including bilingually grown up participants; however, the remainder of the participants could understand and use the German language. Overall, 76.5% (166) of the participants were qualified for university (German “Abitur”), seven had a baccalaureate (“Fachabitur”), 15 participants finished the 10th grade on a German secondary school (“mittlere Reife”) and one finished the German “Hauptschule,” i.e., completed high school. A 1st university degree including one polytechnic degree was held by 27 participants and one holds an advanced technical college certificate. Regarding handedness, 91.7% were right-, 6.9% left-handed and 1.4% stated to use both hands equally. All participants filled in the questionnaires described in the next section and provided a scan of the left and right hand. The study was approved by the local ethic committee of Ulm University, Germany.

### Questionnaires

#### Short Version of Internet Addiction Test (s-IAT)

We collected data on unspecified IA tendencies with the German s-IAT based on the original test by Young (60). The s-IAT consists of two factors, namely loss of control/time management (LoCTM) and craving/social problems (CSP) with six items for each factor (61). All items can be answered on a 5-point-likert scale (1 = “never,” 2 = “rarely,” 3 = “sometimes,” 4 = “often,” and 5 = “very often”) with a possible range from 12 to 60. Scores above 30 until 37 (30 < s-IAT ≤ 37) indicating a slightly increased (problematic) Internet use. Scores over 37 are considered as pathological Internet use. Cronbach’s alpha for the complete questionnaire was α = 0.85 (LoCTM: α_LoCTM_ = 0.79, CSP: α_CSP_ = 0.74).

#### s-IAT Scales

The s-IAT represents five specific problematic Internet use categories—online computer gaming (A1), online gambling (A2), compulsive online buying (A3), online pornography (A4), online social networking (A5). Each category consists out of four items whereas two items each are used to collect data for the factors LoCTM and CSP, respectively. Items to gather information on LoCTM where phrased as follows: “How often do you find that you spend more time with (e.g., online gaming) than you intended?” and “How often do you neglect household chores to spend more time with (e.g., online gaming)?” The following items were used to retrieve information on CSP: “How often do you feel preoccupied with the (e.g., online gaming) when offline, or fantasize about (e.g., online gaming)?” and “How often do you choose to spend more time with (e.g., online gaming) over going out with others?” A 5-point-likert scale (same as in the s-IAT) was used. Cronbach’s alpha for these five scales each consisiting of four anchor items showed the following reliabilities: α_A1_ = 0.87, α_A2_ = 0.64, α_A3_ = 0.77, α_A4_ = 0.83, α_A5_ = 0.79.

### 2D:4D Ratios

To determine 2D:4D ratios, we used scans of both hands. The length of the fingers was measured in pixels (scan resolution 300–400 dpi) starting at the middle of the closest crease to the palm to the fingertip. The measurement was conducted with the graphical software GIMP version 2.8.14.^3^ The 2D:4D ratio was obtained by dividing the length of the index finger by the length of the ring finger. All 2D:4D measurements were executed by two independent raters and then averaged. The reliability of the two raters was high: The interclass correlation coefficients (ICC) with absolute agreement definition of 2D:4D quotients was *ICC*(left) = 0.97 and *ICC*(right) = 0.96. Correlations between the 2D:4D values of the two raters were *r*(left) = 0.93 and *r*(right) = 0.93 (both at *p* < 0.01).

### Statistical Analysis

Statistical analyses were executed with the SPSS version 24.0.0.1 for MAC. Non-parametric testing was applied due to the not normally distributed variables age, s-IAT, and the s-IAT scales. The data of both left and right 2D:4D hand ratios were normally distributed. To identify potential differences between these groups, an independent sample *t*-test was used. We conducted a Mann–Whitney *U* test to check for differences in Internet related variables depending on gender. Spearman correlations were applied to analyze associations between age, Internet variables, and finger ratios.

### Data Cleaning

Since a broken or injured finger can lead to length variations with tremendous influences on the 2D:4D ratio, the following participants were excluded from the sample.

Six participants reported a broken index finger (4 left, 2 right) and seven a broken ring finger (4 left, 3 right). Furthermore, a total of 24 participants reported no information concerning broken fingers that is why we inspected the scans of their hands visually. Since no abnormalities were found, these participants remained in the sample. Moreover, the left index finger of one participants was extremely short (2D:4D ratio = 0.81; 4.9 SD away from mean). Since we did not have any further information rendering a reasonable explanation for this fact, we decided to exclude this participant (only the left hand) from the sample. Consequently, data of *N*_L_ = 208 left 2D:4D ratios and *N*_R_ = 212 right 2D:4D ratios could be used for the analyses. Additionally, one participant did not give information on the s-IAT scale online gaming, which leads to a sample size reduced by 1 for analyses including the specific IA factor online gaming. Moreover, another participant gave no information for the 3rd item of online shopping that is why *N* is reduced by 1 in some tables.

## Results

### Descriptive Statistics and Inferential Statistics for Age, Gender, and Questionnaire Data/Ratios

The means (M) and SD for all questionnaire measures and 2D:4D ratios are presented in Table 1. As depicted in Table 2, we observed higher digit ratios in the right hand in females compared to males [*t*(212) = −2.34, *p* = 0.02]. Gender also influenced several IUD scores (see again Table 1). Among these are higher scores for males compared to females in online gaming (*U* = 2,790, *p* < 0.01), online gambling addiction (*U* = 4,693, *p* < 0.01) and in online pornography addiction (*U* = 2,010, *p* < 0.01). For online communication addiction, females showed higher scores than males (*U* = 4,397, *p* = 0.02). No significant differences were found for s-IAT scores and online shopping addiction.

**Table 1:** Descriptive statistics of age and Internet variable data for the entire sample and split by gender.

	Entire sample *N* = 217						Females *N*(*f*) = 140		Males N(*m*) = 77	
Variables	M (all)	SD (all)	SEM	Min	Max	Skew	M (f)	SD (f)	M (m)	SD (m)
Age	23.41	7.77	0.53	16	63	3.54	22.59	6.73	24.91	9.23
s-IAT	24.88	6.73	0.46	12	57	0.97	24.39	6.31	25.77	7.41
s-IAT_LoCTM_	15.20	4.44	0.30	6	30	0.36	15.11	4.41	15.36	4.51
s-IAT_CSP_	9.67	3.02	0.21	6	27	1.77	9.27	2.50	10.40	3.70

**Cut-off score for problematic Internet use according to Pawlikowski et al. (61): s-IAT > 30 with *N* = 38, *N*(*f*) = 24, *N*(*m*) = 14**										

s-IAT (>30)	35.26	5.54	0.90	31	57	2.35	34.13	3.83	37.21	7.42
s-IAT_LoCTM_ (>30)	21.55	2.85	0.46	16	30	0.59	21.88	1.94	21.00	4.00
s-IAT_CSP_ (>30)	13.71	3.83	0.62	8	27	1.35	12.25	2.71	16.21	4.26

**Cut-off score for unproblematic Internet use according to Pawlikowski et al. (61): s-IAT ≤ 30 with *N* = 179, *N*(*f*) = 116, *N*(*m*) = 63**										

s-IAT (≤30)	22.67	4.55	0.34	12	30	−0.15	22.37	4.61	23.22	4.42
s-IAT_LoCTM_ (≤30)	13.85	3.43	0.26	6	21	−0.05	13.72	3.36	14.11	3.58
s-IAT_CSP_ (≤30)	8.82	1.95	0.15	6	14	0.42	8.66	1.96	9.11	1.92

**Scales A1–A5 assessing different forms of Internet use disorder: *N* = 217, *N*(*f*) = 140, *N*(*m*) = 77**										

A1 s-IAT^a^	5.44	2.60	0.18	4	17	2.28	4.75	1.58	6.97	3.38
A1 s-IAT_LoCTM_^a^	2.85	1.52	0.10	2	10	2.23	2.40	0.88	3.66	2.03
A1 s-IAT_CSP_^a^	2.69	1.25	0.09	2	9	2.35	2.35	0.83	3.31	1.59
A2 s-IAT	4.09	0.40	0.03	4	8	6.18	4.01	0.12	4.22	0.64
A2 s-IAT_LoCTM_	2.03	0.20	0.01	2	4	6.93	2	0	2.09	0.33
A2 s-IAT_CSP_	2.06	0.25	0.02	2	4	4.80	2.01	0.12	2.13	0.38
A3 s-IAT^b^	5.91	2.34	0.16	4	16	1.68	6.06	2.39	5.62	2.23
A3 s-IAT_LoCTM_^b^	3.15	1.49	0.10	2	9	1.49	3.29	1.59	2.91	1.28
A3 s-IAT_CSP_	2.75	1.06	0.07	2	8	1.79	2.77	1.03	2.71	1.12
A4 s-IAT	5.33	2.22	0.15	4	20	2.47	4.42	1.01	6.97	2.81
A4 s-IAT_LoCTM_	2.66	1.28	0.09	2	10	2.32	2.14	0.50	3.62	1.66
A4 s-IAT_CSP_	2.66	1.07	0.07	2	10	2.48	2.29	0.63	3.35	1.35
A5 s-IAT	8.45	3.00	0.20	4	16	0.37	8.76	3.04	7.88	2.85
A5 s-IAT_LoCTM_	4.73	1.90	0.13	2	10	0.39	4.90	1.91	4.43	1.85
A5 s-IAT_CSP_	3.71	1.40	0.10	2	8	0.58	3.86	1.43	3.45	1.32

*M, mean; SD; SEM; s-IAT, short version of the IAT; s-IAT > 30, problematic Internet use starts with values greater than 30 (61), sub-facets of s-IAT: LoCTM, loss of control/time management; CSP, craving/social problems; s-IAT A1: online gaming; s-IAT A2: online gambling; s-IAT A3: online shopping; s-IAT A4: online pornography; s-IAT A5: online communication*.

*^a^*N* is reduced by 1 (one female participant missed to enter their 4 values for A1 online gaming), N = 216, N(f) = 139*.

*^b^*N* is reduced by 1 (one female participant missed to enter the 3rd item of the s-IAT A3 question set: online shopping, A3 s-IAT_CSP_ remains unaffected), N = 216, N(f) = 139*.

**Table 2:** Gender specific differences of hand ratios (*T*-test) and s-IAT scales (Mann–Whitney *U* Test).

2D:4D ratios	Females *N*_L_ = 137, *N*_R_ = 138		Males *N*_L_ = 71, *N*_R_ = 74		*T*-test	
	M	SD	M	SD	*T*-score	*p*
Left hands: *N*_L_ = 208, *df* = 206	0.98	0.03	0.97	0.03	−0.72	0.47
Right hands: *N*_R_ = 212, *df* = 210	**0.98**	0.03	**0.97**	0.03	**−2.34**	**0.02**

IUD-VAR: *N* = 217, *df* = 215	*N*(*f*) = 140 (*N*(*f*) = 139^a,b^)		*N*(*m*) = 77		Mann–Whitney *U*	
	MR		MR		*U*	*p*

Generalized IUD: s-IAT	105.25		115.82		4,865	0.24
Online gaming: s-IAT A1^a^	**90.07**		**141.77**		**2,790**	**<0.01**
Online gambling: s-IAT A2	**104.02**		**118.05**		**4,693**	**<0.01**
Online shopping: s-IAT A3^b^	113.06		100.27		4,718	0.14
Online pornography: s-IAT A4	**84.86**		**152.90**		**2,010**	**<0.01**
Online communication: s-IAT A5	**116.10**		**96.10**		**4,397**	**0.02**

*IUD-VAR, Internet use disorder variable; MR, mean rank; s-IAT A1 to s-IAT A5 scales*.

*^a^*N* is reduced by 1 (one female participant gave no information for online gaming)*.

*^b^*N* is reduced by 1 (one female participant gave no information for the 3rd item of online shopping)*.

Significant findings are displayed in bold letters.

Our results show that age is partially correlated with both right hand ratios and online gaming behavior in females, but not in males. Noteworthy, evidence from literature shows, that 2D:4D markers are stable over life (please see introduction). Nevertheless, we assume that age could be a nuisance variable that might affect some of the following analysis (all correlations are depicted in the Table S2 in Supplementary Material).

### Relationship between Specific Forms of IUD and Unspecified IUD Including Gender Effects

A correlation matrix of all IUD scales shows several significant correlations (Table S4 in Supplementary Material). Of note and in line with an earlier study by Montag et al. (59), the highest and most robust association could be observed between ICD (A5) and general s-IAT score (*rho* = 0.40; *p* < 0.01). The second highest associations could be observed between both Internet Pornography Disorder (IPD, A4) (*rho* = 0.31; *p* < 0.01) and IGD (A1) correlated with unspecified IUD (*rho* = 0.30; *p* < 0.01). Going beyond these findings, it is noticeable that general s-IAT values correlated highest with IGD (A1) in males (*rho*_m,A1_ = 0.52, *p* < 0.01) and with ICD (A5) in females (*rho*_f,A5_ = 0.48, *p* < 0.01). Therefore, we consider gender as an important variable to get more detailed information about these associations. Please note, that the trend-results remained unchanged when using partialized correlations controlling for age. Only in males, Internet Shopping Disorder (ISD, A3) (*r* = 0.39, *p* < 0.01) correlated significantly and higher than in usual bivariate correlations with general s-IAT (Table S5 in Supplementary Material).

### 2D:4D Ratios in Reference to Unspecified IUD (s-IAT) Online Gaming (A1), Online Gambling (A2), and Online Pornography (A4) in Left/Right Hands

We found an inverse association between the finger ratios in right male hands and the sub-facet LoCTM of the s-IAT (*rho*_R_ = −0.24, *p* = 0.04, *N*_R,m_ = 74) and a negative correlation independent of gender between the 2D:4D ratio of the right hand and IGD (A1) (*rho*_R_ = −0.17, *p* = 0.01, *N* = 211). The effect is driven by the facet of loss of control of Internet Gaming in the whole sample (*rho* = −0.20, *p* < 0.01, *N* = 211) and the sub-facet LoCTM in the female sample [*rho*_R_(LoCTM) = −0.20, *p* = 0.02, *N* = 137]. For online gambling, we discovered a negative association (*rho*_R_ = −0.17, *p* = 0.01, *N* = 212) driven by the sub-facet CSP in the female sample (*rho*_R_ = −0.17, *p* = 0.05, *N* = 138).

IPD in the entire sample also negatively associates to right hand finger ratios (*rho*_R_ = −0.16, *p* = 0.02, *N* = 212, A4) triggered by both sub-facets (*rho*_R_(LoCTM) = −0.15, *p* = 0.03, *N*_R,m_ = 212, *rho*_R_(CSR) = −0.15, *p* = 0.03, *N*_R,m_ = 212) but could not be found in male or female subsamples. No significant associations for the left hand could be observed (further results are depicted in Table 3). Please note that we do not control for age in the presented analysis, because IGD was not associated with age in the complete sample.

**Table 3:** Correlations of left and right hand ratios with Internet addiction variables including gender effects (Spearman’s *rho*).

	2D:4D LEFT hands			2D:4D RIGHT hands		
	Entire sample *N*_L_ = 208	Females *N*(*f*)_L_ = 137	Males *N*(*m*)_L_ = 71	Entire sample *N*_R_ = 212	Females *N*(*f*)_R_ = 138	Males *N*(*m*)_R_ = 74
s-IAT	−0.01	−0.02	0.01	−0.04	0.05	−0.19
s-IAT_LoCTM_	−0.02	−0.03	−0.01	−0.05	0.05	−0.24*
s-IAT_CSP_	0.03	0.02	0.06	−0.01	0.02	−0.03
**Online gaming: A1 s-IAT^a^**	−0.10^a^	−0.11^a^	−0.06	−0.17*^,a^	−0.17*^,a^	−0.04
A1 s-IAT_LoCTM_^a^	−0.10^a^	−0.15^a^	−0.01	−0.20**^,a^	−0.20*^,a^	−0.10
A1 s-IAT_CSP_^a^	−0.07^a^	−0.05^a^	−0.06	−0.11^a^	−0.10^a^	0.02
**Online gambling: A2 s-IAT**	−0.11	−0.14	−0.07	−0.17*	−0.17*^,d^	−0.13
A2 s-IAT_LoCTM_^c^	0.01	^c^n.a.	0.04	−0.09	^c^n.a.	−0.08
A2 s-IAT_CSP_	−0.11	−0.14	−0.10	−0.16*	−0.17*^,d^	−0.13
**Online shopping: A3 s-IAT^b^**	0.07^b^	0.09^b^	−0.01	0.06^b^	0.12^b^	−0.16
A3 s-IAT_LoCTM_^b^	0.11^b^	0.11^b^	0.07	0.09^b^	0.13^b^	−0.11
A3 s-IAT_CSP_	0.00	0.03	−0.08	−0.01	0.04	−0.15
**Online pornography: A4 s-IAT**	−0.05	−0.08	0.08	−0.16*	−0.11	−0.05
A4 s-IAT_LoCTM_	−0.04	−0.11	0.05	−0.15*	−0.15	−0.03
A4 s-IAT_CSP_	−0.02	−0.05	0.13	−0.15*	−0.11	−0.03
**Online communication: A5 s-IAT**	0.02	0.01	−0.01	−0.02	0.04	−0.20
A5 s-IAT_LoCTM_	0.01	−0.02	0.04	−0.03	0.03	−0.20
A5 s-IAT_CSP_	0.01	0.05	−0.08	−0.02	0.03	−0.18

Correlation (2-tailed) is significant at the 0.01 level (**), at the 0.05 level (*); sub-facets of s-IAT: LoCTM, loss of control/time management; CSP, craving/social problems; s-IAT A1 to s-IAT A5 scales.

*^a^*N* is reduced by 1 (one female participant did not provide information for online gaming): for LEFT hands N_L_ = 207, N(f)_L_ = 136; for RIGHT hands N_R_ = 211, N(f)_R_ = 137*.

*^b^*N* is reduced by 1 (one female participant did not provide information for the 3rd item of online shopping, A3 s-IAT_CSP_ remains unaffected): for LEFT hands N_L_ = 207, N(f)_L_ = 136; for RIGHT hands N_R_ = 211, N(f)_R_ = 137*.

*^c^All female participants chose the lowest value: M = 2, SD = 0*.

*^d^p = 0.05*.

*Significant findings are displayed in bold letters*.

The right 2D:4D ratio of females was inversely correlated with IGD symptoms in the aforementioned sub-facet [*rho*_R_(LoCTM) = −0.20, *p* = 0.02, *N* = 137]. LoCTM was not associated with age on the right-hand side. For online gambling, the sub-facet CSP is also associated with 2D:4D [*rho*_R_ = *rho*_R_(CSP) = −0.17, *p* = 0.01, *N* = 138]. Due to the significant association between age and craving for online gambling, we controlled for age (for further results, please refer to Table 3 and Table S2 in Supplementary Material).

Given that none of the associations beyond unspecified IUD/IGD were hypothesized (in terms of earlier existing works on 2D:4D and different forms of IUD), these results would not hold for multiple testing. Nevertheless, we present them for future research endeavors.

## Discussion

Before coming to the first hypothesis, we revisit the question if in particular female hands show higher digit ratios than male ones. We could find such a significant difference in the right hand of our participants, which is in line with what has been observed most robustly in the literature (30). To further check if our collected data set is valid in the context of the investigation of IUD data, we tested in the result section often observed gender differences on the unspecified IUD score and the specific IUD scores as summarized in the introduction. For IUD in the areas of gaming, gambling and pornography usage, we replicated the well-known findings that males show significant higher scores. In contrast, females showed significantly higher scores in ICD than males. For IUD in the area of online shopping, no significant differences could be observed, although descriptive statistics point toward the observed findings in the literature with higher scores in females toward males. In sum, the findings both with respect to the present 2D:4D ratios and IUD data are largely in line with what has been presented in the research field before and demonstrates the validity of our data.

### 2D:4D Ratios and Tendencies toward IGD

The main focus of our study was to revisit the association between lower 2D:4D markers and higher IGD tendencies as observed in the literature in males. The studies in the field by Kornhuber et al. (58) and Canan et al. (57) both observed lower 2D:4D ratios (hence higher prenatal testosterone levels) to be associated with higher tendencies toward IUD in males. To be more precise, Kornhuber et al. (58) linked lower 2D:4D ratios to higher video game addiction when contrasting *N* = 27 online gaming addicts with *N* = 27 healthy controls. They used the *CSAS II* measure for assessing and classifying video gaming addiction. The authors report that “CSAS II is based on the Internet Addiction Scale ISS-20, which has been extended and adapted to assess video game addiction” (p. 2). In Canan et al. (57), Young’s 20 item IAT was administered to assess unspecified IUD. Furthermore, as the authors did not assess IUD tendencies in other specific online areas, nevertheless they asked for frequent online activities of the study’s participants and split the IUD scores (testing generalized IUD) in subsamples according to the most frequented online channel of each user. For example, this resulted in N = 55 out of *N* = 650/652 persons who reported to frequently spent time online for gaming. The unspecified IUD scores for these 55 participants were then compared to the unspecified IUD scores of the subsample reporting social network use (*n* = 315) and streaming videos (*n* = 206) and others (*n* = 74). The resulting statistic in Canan et al. (57) underlined that the observed lower 2D:4D markers in males with higher unspecified IUD might be accountable for overusage in the online gaming niche.

Our study goes beyond the described published findings, because (a) we assessed IUD in five different specific domains (gaming, gambling, shopping, pornography, communication) as well as unspecified IUD. Furthermore, and (b) the present work did not use a cut-off to search for subgroups of addicted or non-addicted persons. Instead we searched for continual associations between healthy and pathological usage of the Internet (and specific online channels) in the complete investigated sample of *N* = 217 participants. At first glance, our findings are in line with what has been observed in the works by Kornhuber et al. (58) and Canan et al. (57), namely that lower 2D:4D ratios of the right hand are associated with higher IGD. However, we also observed that this correlation was driven by female participants for the right hand and it could not be found in males. This is surprising, because earlier studies observed this effect exclusively in males.

In order to find an explanation for this occurrence, we compare our study’s participants with those from the earlier published research. First of all, both of us, found the same associations with respect to the right digit hand ratios and IUD questionnaire. This speaks against the idea, that our sample would be somewhat different from what has been observed in the literature. The Kornhuber et al. study only investigated (video game addicted vs. healthy) males and naturally their sample is hard to compare with the participants from the present work. Canan et al. [(57), p. 32] report for their sample, that “Women were more likely to use the Internet for social networking and streaming videos than were men. Compared to women, a higher proportion of men reported Internet gaming as their most frequent Internet activity (*p* < 0.001)”. In light of this, we believe that our samples do not strongly differ in these areas. Asides, we assessed addictive tendencies in the “Big Five of IUD” as mentioned above, whereas the Canan et al. (57) study only assessed in what areas the Internet usually is used (no addictive tendencies have been directly assessed here and the categories investigated only overlap in parts). Therefore, our studies are only comparable to a certain extent. Moreover, and of importance, the facet loss of control of generalized IUD was inversely associated with right 2D:4D ratios in the present male sample supporting the findings associating the digit ratio with generalized IUD as reported by Canan et al. (57) to some extent.

Our findings suggest that further reasons might be considered for not observing the association between digit ratios and IGD in males, but only females. First, the study by Canan et al. (57) has been conducted in Turkey. As our study stems from Germany, cultural differences could account for the present results. These factors might include differences in personality, where differences between Turkish and German samples have been observed (62). In this study, Western Europeans (including Germany) scored lower on agreeableness and conscientiousness compared to Middle Eastern countries (including Turkey). Such differences could influence associations as observed in the present work, because the 2D:4D marker has been also associated with personality (43, 44). In addition, our present study only included a relatively small group of males (*n* = 77 males vs. *n* = 140 females), because we recruited the present sample mainly in psychological classes. Therefore, our statistical power to detect the same effects for males as for females is smaller. In contrast to the power hypothesis, there is a low observed correlation between right hand digit ratio and IGD in the male group of participants in Table 3: *rho* = −0.04, *p* = 0.72, *N* = 74. In sum, the digit ratio of the right hand might not be exclusively linked to IGD in males (57, 58), but can be linked to IGD in females. Therefore, female participants being under stronger influence of prenatal testosterone (or prenatal testosterone to estradiol level) might also show more male online behavior in terms of IGD. Developing from the present results, future studies clearly should recruit females to investigate putative links between IGD and the digit ratio of the (right) hand. Again, we state that when assessing generalized IUD in our male participants, more similar findings can be observed to what has been reported by Canan et al. (57).

Beyond this, no further robust association could be observed with any other specific area of IUD. Note, that the associations between 2D:4D ratio and tendencies toward overusage of online gambling or pornography will not be further discussed, because they would not hold for multiple testing given the lack of set up á priori hypothesis (although we set up directed hypothesis, only empiricial evidence was existing on IGD/unspecified IUD and 2D:4D before our study). This underlines that prenatal testosterone might be exclusively linked to IGD/unspecified IUD but not to any other areas when assessing IUD in different contexts. Nevertheless, this statement might be revised, when other researchers also observe our associations between 2D:4D and gambling/pornography usage. It needs to be remembered that the present study investigated a largely healthy student sample population (and the scores on the IUD questionnaires where highly skewed toward the lower end of the distribution). In particular with the sex-dimorphisms observed in many areas of online usage, clinical samples might reveal different outcomes.

### Revisiting the Question on the Link between Unspecified IUD and Specific Forms of IUD under Consideration of Gender

The last aim of our study was to revisit findings from an earlier study by Montag et al. (59) observing in six independent samples from diverse cultural background that ICD is most strongly linked with unspecified IUD. This earlier study could not consider gender as an independent variable (although assessed), because the samples differed strongly in gender ratios, e.g., one of the investigated samples only had 9 males and 66 females (Germany, paper-pencil sample) or other, more well distributed samples (23 males and 28 females; China, paper-pencil sample), were still too small to search for robust gender differences with respect to the IUD correlations. The only sample from this earlier study, in which such an association could have been examined stemming from China (online sample), but it was generally not in the realm of this earlier work given the many differences in the socio-demographics across all samples investigated. Therefore, we return to this question in the present work. For the complete sample, we could replicate the finding that tendencies toward ICD are most strongly associated with unspecified IUD (*r* = 0.40**, see Table S5 in Supplementary Material). Splitting these results into a male and female subsample reveals that this association is driven by the female subsample (*r* = 0.50**), whereas in males it is weaker (*r* = 0.29**). The second and third strongest association between specific forms and unspecified IUD was shopping (*r* = 0.34**) and pornography (*r* = 0.28**) in females. In males, significant associations occurred most prominently with gaming (*r* = 0.57**), pornography (*r* = 0.53**) and shopping (*r* = 0.39**). Perhaps except for the pornography-unspecified IUD link, the associations are in line with the gender differences observed in the literature.

Our new data clearly show that association patterns between specific forms of IUD and unspecified IUD need to be investigated also in the context of males and females. This said, the general observed robust association between ICD and unspecified IUD by Montag et al. (59) is supported in the present finding. It needs to be mentioned that a large sample stemming from China including much more males than females (281 vs. 63) also observed strong associations between tendencies toward ICD and unspecified IUD [complete sample: *rho* = 0.68, *p* < 0.01; males: *rho* = 0.67, *p* < 0.01; females: *rho* = 0.70, *p* < 0.01, (59)]. Finally, the measures to assess the different forms of specific Internet addictions differed between the present and our earlier work. Clearly, the associations between specific forms of IUD and unspecified IUD are complicated and simple correlations analysis need to be enhanced by moderation/mediation analysis in the near future taking into account variables such as Internet Use Expectancies as well as Gratification and Compensation Processes for each domain [aside from the already mentioned gender and cultural dimensions; see also Ref. (3)].

## Conclusion

The present study found further evidence for a role of the 2D:4D marker of the right hand in IGD. Our findings indicate that lower digit ratios (hence higher prenatal testosterone levels) are associated with higher tendencies toward IGD. Noteworthy, our findings could only be observed in females and not males, something, which could only be partly explained (probably a higher *n* of males is warranted and/or more afflicted male persons compared to the subclinical sample investigated in the present work). In males, a negative association appeared between the digit ratio of the right hand and generalized IUD (facet loss of control) supporting the findings of Canan et al. (57). Future studies need to include even larger, more gender-balanced samples to investigate the rather small (but also robust) associations between the 2D:4D marker and IGD.

## Ethics Statement

The study was approved by the local ethic committee of Ulm University, Germany. Under https://www.uni-ulm.de/einrichtungen/ethikkommission-der-universitaet-ulm/ the official website of the ethic committee can be found.

## Author Contributions

CM, MM, and MB designed the study. MM carried out the statistical analysis. MM drafted the method and result section (and supplement). All statistical analyses were checked by BL. MM and JM carried out the measurement of the hands. CM wrote the first draft of the introduction. CM and MM wrote the first draft of the discussion. MB, RS, and BL critically revised the manuscript.

## Conflict of Interest Statement

The authors declare that the research was conducted in the absence of any commercial or financial relationships that could be construed as a potential conflict of interest.

## Footnotes

^1^http://www.internetlivestats.com (accessed on 14th of October 2017).

^2^In the following, we speak of *unspecified* instead of generalized IUD, because a person filling in a measure for generalized IUD might implicitly think about his/her most prevalent online channel(s).

^3^http://www.gimp.org.
