# Supplementary material for: The 2D:4D Marker and Different Forms of Internet Use Disorder
Source: Front Psychiatry. 2017 Nov 20;8:213. doi: 10.3389/fpsyt.2017.00213 (PMC5715388; doi:10.3389/fpsyt.2017.00213)
Supplement: Supplementary file 1 [file table_1.pdf]

frontiers

Supplementary Material

Table 1 | Descriptive hand ratio data of the entire sample and split by gender

	Entire sample $N_L = 208$, $N_R = 212$						Females $n_L = 137$, $n_R = 138$		Males $n_L = 71$, $n_R = 74$	
**variables**	**M(all)**	**SD(all)**	**SEM**	**Min**	**Max**	**Skew**	**M(f)**	**SD(f)**	**M(m)**	**SD(m)**
LEFT 2D:4D ratio	0.98	0.03	0.00	0.89	1.07	0.05	0.98	0.03	0.97	0.03
RIGHT 2D:4D ratio	0.97	0.03	0.00	0.90	1.05	0.04	0.98	0.03	0.97	0.03

Note. M (Mean), SD (Standard Deviation), SEM (Standard Error of the Mean)

Table 2 | Age correlated with both hand ratios and Internet addiction variables for the entire sample and both genders using Spearman's *rho*

	**2D:4D LEFT hands**			**2D:4D RIGHT hands**		
	**Entire sample** $N_L = 208$	**Females** $n_L = 137$	**Males** $n_L = 71$	**Entire sample** $N_R = 212$	**Females** $n_R = 138$	**Males** $n_R = 74$
age	23.27 ±7.50	22.61 ±6.80	24.55 ±8.61	23.45 ±7.85	22.63 ±6.77	24.99 ±9.40
2D:4D hands ratios	-0.03	0.06	-0.15	0.05	**0.19***	-0.10
s-IAT	-0.13	-0.14	-0.18	-0.10	-0.11	-0.15
s-IAT$_{LoCTM}$	**-0.19****	**-0.19***	**-0.24***	**-0.17***	-0.17	**-0.23***[d]
s-IAT$_{CSP}$	0.02	0.02	-0.04	0.04	0.02	0.00
[a]A1 s-IAT	-0.06	**-0.17***	-0.16	-0.06	**-0.17***	-0.15
[a]A1 s-IAT$_{LoCTM}$	-0.07	**-0.18***	-0.13	-0.05	-0.16	-0.11
[a]A1 s-IAT$_{CSP}$	-0.04	-0.13	-0.15	-0.05	-0.13	-0.16
A2 s-IAT	0.10	0.05	0.08	0.10	0.05	0.07
[c]A2 s-IAT$_{LoCTM}$	0.05	[c]n.v.	0.03	0.05	[c]n.v.	0.03
A2 s-IAT$_{CSP}$	0.06	0.05	0.01	0.05	0.05	0.00
[b]A3 s-IAT	0.01	-0.07	0.17	0.01	-0.07	0.17
[b]A3 s-IAT$_{LoCTM}$	0.04	-0.01	0.19	0.04	-0.02	0.19
A3 s-IAT$_{CSP}$	-0.01	-0.10	0.18	0.00	-0.09	0.16
A4 s-IAT	-0.01	-0.16 ($p = 0.06$)	-0.10	0.01	**-0.17***	-0.04
A4 s-IAT$_{LoCTM}$	0.07	-0.04	-0.06	0.09	-0.05	-0.01
A4 s-IAT$_{CSP}$	-0.05	**-0.19***	-0.12	-0.03	**-0.20***	-0.05
A5 s-IAT	**-0.19****	**-0.21***	-0.13	**-0.18****	**-0.20***	-0.12
A5 s-IAT$_{LoCTM}$	**-0.20****	**-0.22***	-0.18	**-0.19****	**-0.21***	-0.16
A5 s-IAT$_{CSP}$	-0.12	-0.16	-0.01	-0.13	-0.15	-0.04

Note. Correlation (2-tailed) is significant at the 0.01 level (**), at the 0.05 level (*); sub-facets of s-IAT: LoCTM (loss of control/time management), CSP (craving/social problems), s-IAT A1: online gaming, s-IAT A2: online gambling, s-IAT A3: online shopping, s-IAT A4: for online pornography, s-IAT A5: online communication.

[a]*N* is reduced by 1 (one participant missed to enter their 4 values for A1 online gaming): for LEFT hands $N_L = 207$, $N(f)_L = 136$; for RIGHT hands $N_R = 211$, $N(f)_R = 137$

[b]*N* is reduced by 1 (one participant missed to enter the 3rd item of the s-IAT A3 question set: online shopping, A3 s-IAT$_{CSP}$ remains unaffected): for LEFT hands $N_L = 207$, $N(f)_L = 136$; for RIGHT hands $N_R = 211$, $N(f)_R = 137$

[c]all female participants chose the lowest value: $M = 2$, $SD = 0$

[d]$p = 0.05$

Table 3 | Spearman's *rho* of age correlated with each item of the s-IAT and the five scales (A1-A5) for the entire sample and both genders

**s-IAT item no.**	**1**	**2**	**3**	**4**	**5**	**6**	**7**	**8**	**9**	**10**	**11**	**12**
All (*N* = 217)	-0.10	-0.13	-0.12	0.09	0.04	-0.06	-0.05	-0.12	**-0.18****	0.02	0.08	0.03
Female (*n* = 140)	-0.15	-0.16	-0.06	0.10	-0.02	-0.05	-0.08	**-0.18***	**-0.17***	0.02	0.04	-0.01
Male (*n* = 77)	-0.02	-0.14	**-0.30****	-0.01	0.07	-0.06	-0.05	-0.09	-0.19	0.00	0.10	-0.02
**Scales**	**A1-1**	**A1-2**	**A1-3**	**A1-4**	**A2-1**	**A2-2**	**A2-3**	**A2-4**	**A3-1**	**A3-2**	**A3-3**	**A3-4**
All (*N* = 217)	[a]-0.03	[a] -0.02	[a] -0.01	[a] -0.04	0.05	0.07	0.08	0.02	0.03	0.02	0.10	-0.01
Female (*n* = 140)	[a] -0.15	[a] -0.09	[a] -0.008	[a] -0.06	[c]n.v.	0.05	[c]n.v.	[c]n.v.	-0.04	-0.08	[b]0.03	-0.09
Male (*n* = 77)	-0.10	-0.12	-0.07	-0.17	0.02	0.03	0.10	0.00	0.16	0.21	**0.25***	0.14
**Scales**	**A4-1**	**A4-2**	**A4-3**	**A4-4**	**A5-1**	**A5-2**	**A5-3**	**A5-4**				
All (*N* = 217)	0.10	-0.02	0.06	0.06	**-0.16***	**-0.17****	**-0.18****	0.00				
Female (*n* = 140)	-0.03	**-0.19***	0.01	0.01	**-0.17***	**-0.22****	**-0.22****	-0.01				
Male (*n* = 77)	0.01	-0.05	-0.05	0.02	-0.11	-0.06	-0.11	0.06				

Note. Correlation (2-tailed) is significant at the 0.01 level (**), at the 0.05 level (*); greyish fields account for the sub facet LoCTM, white background for the sub facet CSP, [a]*N* is reduced by 1 (one participant missed to enter their 4 values for A1 online gaming), [b]*N* is reduced by 1 (one participant missed to enter the 3rd item of the s-IAT A3 question set: online shopping), [c]all female participants chose the lowest value: $M = 2$, $SD = 0$

Table 4 | Spearman's *rhos* between all IUD variables (s-IAT and scales A1 - A5) for the entire sample and both genders

**IUD-VAR**	**Entire sample (*N* = 217, [a,b]*N* = 216)**				
	s-IAT	[a]A1 s-IAT	A2 s-IAT	[b]A3 s-IAT	A4 s-IAT
[a]A1 s-IAT	**0.30****				
A2 s-IAT	-0.01	**0.17***			
[b]A3 s-IAT	**0.19****	0.11	-0.03		
A4 s-IAT	**0.31****	**0.33****	**0.17***	0.13[c]	
A5 s-IAT	**0.40****	0.04	0.01	**0.33****	0.11

**IUD-VAR**	**Females (*n* = 140, [a,b]*n* = 139)**					**Males (*n* = 77)**				
	s-IAT	[a]A1 s-IAT	A2 s-IAT	[b]A3 s-IAT	A4 s-IAT	s-IAT	A1 s-IAT	A2 s-IAT	A3 s-IAT	A4 s-IAT
[a]A1 s-IAT	0.15					**0.52****				
A2 s-IAT	-0.02	**0.18***				-0.07	0.03			
[b]A3 s-IAT	**0.28****	**0.22****	-0.03			0.03	0.07	0.03		
A4 s-IAT	**0.28****	0.10	0.10	**0.21***		**0.39****	0.17	-0.08	**0.27***	
A5 s-IAT	**0.48****	0.04	-0.08	**0.21***	**0.19***	**0.28***	0.20	0.12	**0.49****	**0.40****

Note. IUD-VAR (Internet use disorder variable), Correlation (2-tailed) is significant at the 0.01 level (**), at the 0.05 level (*); s-IAT A1: online gaming, s-IAT A2: online gambling, s-IAT A3: online shopping, s-IAT A4: for online pornography, s-IAT A5: online communication.
[a]*N* is reduced by 1 (one participant missed to enter their 4 values for A1 online gaming)
[b]*N* is reduced by 1 (one participant missed to enter the 3rd item of the A3 s-IAT question set)
[c]$p = 0.05$

Table 5 | Partial correlation coefficients corrected by age between all IUD variables for the entire sample and both genders

IUD-VAR	Entire sample (*df* = 212)				
	s-IAT	[a]A1 s-IAT	A2 s-IAT	[b]A3 s-IAT	A4 s-IAT
[a]A1 s-IAT	**0.39****				
A2 s-IAT	0.00	**0.17***			
[b]A3 s-IAT	**0.34****	**0.19****	-0.03		
A4 s-IAT	**0.39****	**0.41****	0.07	**0.20****	
A5 s-IAT	**0.40****	0.04	0.11	**0.34****	0.11

IUD-VAR	Females (*df* = 135)					Males (*df* = 74)				
	s-IAT	[a]A1 s-IAT	A2 s-IAT	[b]A3 s-IAT	A4 s-IAT	s-IAT	A1 s-IAT	A2 s-IAT	A3 s-IAT	A4 s-IAT
[a]A1 s-IAT	0.16					**0.57****				
A2 s-IAT	-0.02	0.10				-0.04	0.07			
[b]A3 s-IAT	**0.34****	**0.32****	-0.05			**0.39****	**0.24***	0.01		
A4 s-IAT	**0.28****	0.02	**0.19***	0.13		**0.53****	**0.30****	-0.13	**0.51****	
A5 s-IAT	**0.50****	0.07	-0.07	**0.25****	**0.21***	**0.29***	0.16	**0.30****	**0.51****	**0.27***

Note. IUD-VAR (Internet use disorder variable), Correlation (2-tailed) is significant at the 0.01 level (**), at the 0.05 level (*);s-IAT A1: online gaming, s-IAT A2: online gambling, s-IAT A3: online shopping, s-IAT A4: for online pornography, s-IAT A5: online communication.
[a]*N* is reduced by 1 (one participant missed to enter their 4 values for A1 online gaming)
[b]*N* is reduced by 1 (one participant missed to enter the 3rd item of the A3 s-IAT question set)

A test for differences of finger ratios regarding the s-IAT cut-off value (30) stated in Pawlikowski et al. (2013) between both problematic (> 30) and unproblematic (≤ 30) Internet user groups. T-tests between the hands in the entire sample and both genders, respectively, did not reveal any differences (Table 6).

Table 6 | Differences of hand ratios between problematic and ordinary Internet user groups

2D:4D ratios	Problematic Internet use: s-IAT > 30		Normal Internet use: s-IAT ≤ 30		T-test	
	M	*SD*	*M*	*SD*	*T-score*	*p*
**Left hands:** $N_L = 208$, $n(> 30) = 36$, $n(\leq 30) = 172$	0.97	0.03	0.98	0.03	-0.28	.078
**Females**: $n_L = 137$, $n(> 30) = 23$, $n(\leq 30) = 114$	0.97	0.03	0.98	0.03	-0.62	0.54
**Males**: $n_L = 71$, $n(> 30) = 13$, $n(\leq 30) = 58$	0.98	0.03	0.97	0.03	0.43	0.67
**Right hands:** $N_R = 212$, $n(> 30) = 35$, $n(\leq 30) = 177$	0.97	0.04	0.97	0.03	-1.26	0.21
**Females**: $n = 138$, $n(> 30) = 23$, $n(\leq 30) = 115$	0.97	0.04	0.98	0.03	-0.56	0.58
**Males**: $n = 74$, $n(> 30) = 12$, $n(\leq 30) = 62$	0.95	0.03	0.97	0.03	-1.49	0.14
